# Reducing loneliness and depressive symptoms in older adults during the COVID-19 pandemic: A pre-post evaluation of a psychosocial online intervention

**DOI:** 10.1371/journal.pone.0311883

**Published:** 2024-12-13

**Authors:** Aina Gabarrell-Pascuet, Laura Coll-Planas, Sergi Blancafort Alias, Regina Martínez Pascual, Josep Maria Haro, Joan Domènech-Abella

**Affiliations:** 1 Epidemiology of Mental Health Disorders and Ageing Research Group, Sant Joan de Déu Research Institute, Barcelona, Esplugues de Llobregat, Spain; 2 Research, Teaching, and Innovation Unit, Parc Sanitari Sant Joan de Déu, Sant Boi de Llobregat, Spain; 3 Centro de Investigación Biomédica en Red de Salud Mental (CIBERSAM), Instituto de Salud Carlos III, Madrid, Spain; 4 Department of Medicine, Universitat de Barcelona, Barcelona, Spain; 5 Research group on Methodology, Methods, Models and Outcomes of Health and Social Sciences (M3O), Faculty of Health Sciences and Welfare, Centre for Health and Social Care Research (CESS), University of Vic-Central University of Catalonia (UVic-UCC), Vic, Barcelona, Spain; 6 Institute for Research and Innovation in Life Sciences and Health in Central Catalonia (IRIS-CC), Vic, Spain; 7 Fundació Salut i Envelliment UAB, Universitat Autònoma de Barcelona, Barcelona, Spain; Kırklareli University: Kirklareli Universitesi, TÜRKIYE

## Abstract

**Background:**

Loneliness is related to worse mental health, particularly in people with poor social support. The COVID-19 pandemic altered our lives and ways of social interaction, especially among vulnerable populations such as older adults.

**Methods:**

We designed a group-based psychosocial online intervention for older adults (≥ 65 years) facilitated by gerontologists addressing loneliness consisting of: (i) sharing experiences and promoting peer support to overcome feelings of loneliness and (ii) increasing the chances of establishing successful social relationships. This was a feasibility non-controlled prospective pilot study carried out during the COVID-19 pandemic with a pre-post evaluation. Interviews before and after the intervention assessed loneliness (emotional and social), social support, depressive and anxiety symptoms, quality of life, and perceived health. Groups of 6–8 participants and 2 facilitators met once a week for 8 weeks through videoconferencing. The intervention effectiveness was assessed with multilevel models for repeated measures.

**Results:**

The study sample (N = 27) was mainly composed of females (74%) and the mean age was 74.26 years. 21 participants completed the intervention (22% drop-out rate). Statistically significant (*p*<0.01) decreases in emotional loneliness and depressive symptoms were observed following the intervention. Qualitatively, participants positively evaluated the intervention and found in the group a space for personal growth where they could meet new people and express themselves with confidence and security.

**Conclusions:**

Interventions overcoming social distancing restrictions through online tools and targeting vulnerable population sectors (e.g., older adults) can become essential to lessen the collateral consequences of the COVID-19 pandemic on social behaviour and mental health.

## Introduction

Loneliness is a feeling experienced when the quantity and quality of our social relationships do not meet our expectations [1]. It is a subjective feeling that does not necessarily relate to the number of people surrounding us. According to Weiss [2], loneliness can be considered a multifaceted construct comprised of a social and an emotional dimension. Social loneliness emerges from the absence of an available and satisfying social network that can provide a sense of belonging and is connected to social factors such as having close friendships, companionship, and the size of one’s social network. Emotional loneliness refers to the absence of one or more attachment figures with whom one could establish a close connection, and the desired level of intimacy or confidence is not achieved.

Being lonely has been associated with all-cause mortality [3], higher rates of morbidity, worse physical health [4], a faster rate of cognitive decline [5], and impaired functional status and quality of life [6]. Loneliness is related to increases in anxiety and depressive symptomatology (and their comorbidities), particularly in people with poor social support [7, 8]. In recent years, we have witnessed an increasing prevalence of loneliness, with nearly a third of individuals in developed countries experiencing its impact [9]. According to a recent meta-analysis including data from 2000 to 2019, loneliness prevalence in Europe ranges from 5.3% in young adults to 11.9% in older adults [10].

A previous longitudinal study with a 7-year follow-up performed by our research team, identified social support and loneliness as potential targets in people with major depressive disorder (MDD). We reported that lower social support predicted higher subsequent levels of loneliness, which in turn predicted higher probabilities of MDD in a sample of older adults (50 years or older) with MDD at baseline [11]. Moreover, having a small social network has a negative impact on depression in lonely people [12], so increasing social support by creating opportunities for successful social interactions may reduce depressive symptomatology [13, 14].

The effect of socially disruptive measures on social relationships in the context of the COVID-19 pandemic increased feelings of loneliness [15, 16] and the prevalence of mental health problems among older adults [17, 18]. In addition, aging can be accompanied by events that can limit social participation, such as the loss of people from our social environment, retirement, and health problems. This vital transition implies a personal and social adaptation of the individual to a new social role or personal situation, being a stressful moment that can lead to non-desired loneliness [19].

The highly prevalent late-life loneliness [20], accompanied by its adverse health effects, calls for a heightened focus on the development of effective interventions to address this escalating public health issue. It is important that interventions offer new social opportunities while also prompting a shift in how individuals approach and perceive social relationships on a broader scale. Moreover, such interventions must be adapted to the social restrictions needed to contain the spread of COVID-19 pandemic, which highlighted the need to explore remote delivery methods. The adaptation of these interventions is not only relevant in the pandemic context but can also be employed in future similar situations or for individuals unable to physically travel to the intervention site (e.g., due to distance or mobility issues). Additionally, online interactions enable better reconciliation with daily activities and can also help facilitate initial contacts for individuals who have difficulties establishing social relationships, especially in face-to-face settings.

Considering the different targets we can address to reduce loneliness, there are mainly four types of interventions. We can target the community or social support level by (1) enhancing social skills, (2) providing social support, or (3) increasing opportunities for social interaction. Additionally, interventions can target the individual or psychological level by (4) addressing maladaptative social cognition (e.g., cognitive behavioural therapy to identify and reframe negative perceptions and thoughts of loneliness) [21]. Cohen-Mansfield & Perach [22] concluded in their review that combining multiple approaches seems to be the most promising strategy to reduce loneliness.

The most effective tools to deliver interventions targeting social support and aimed at alleviating loneliness are group-based interventions with educational inputs or support activities for specific groups of older people and with the presence of facilitators who encourage the participation of participants in decision-making [23–25]. However, the studies that applied it used heterogeneous health measures, obtaining both positive and negative results [26, 27]. Thus, the health effects of loneliness interventions are promising but inconclusive to date.

Likewise, systematic reviews on interventions to reduce loneliness that include online interventions [28, 29] suggest that new technologies can be considered a promising tool, but although most interventions report some effectiveness in reducing social isolation and loneliness, the quality of the evidence is generally weak. According to a recent scoping review of reviews [30], it is crucial to acknowledge that there is no universal approach to addressing loneliness and, as a result, interventions should be tailored to meet the unique needs of each participant. In this regard, modular interventions offer greater flexibility to adapt to the specific needs of each participant.

In the pandemic context, studies based on previous research and using a rigorous methodology were needed, so we designed a psychosocial online intervention following the assumption that interventions addressing loneliness to improve mental health should follow a modular structure and have a dual focus: (i) sharing experiences and promoting peer support to overcome feelings of loneliness and (ii) increasing the chances of establishing successful social relationships.

The design of the intervention took into account previous programs with similar aims that showed promising results in psychosocial well-being (including mental health, social support, and loneliness), such as the "Circle of Friends" [31, 32], conducted in Finland, and in the Spanish context "Paths: from loneliness to participation" [27, 33] and "Feeling good" [34]. These programs were conducted through face-to-face group sessions. In the present project, its main components were adapted to an online format.

Therefore, we aim to assess the feasibility of an online psychosocial group-based pilot intervention named “Breaking Loneliness, Opening Community” (BLOC). The intervention followed a pre-post evaluation design aimed at testing the following hypotheses: 1) the intervention has a positive effect on participants’ feelings of loneliness, and 2) it contributes to improving social support, symptoms of depression and anxiety, quality of life, and self-reported health of the participants.

## Methods

### Study design and setting

We conducted a non-controlled prospective pilot study with a pre-post evaluation. The study was performed from October 2021 to January 2022 in Barcelona (Spain), during the COVID-19 pandemic. During the study, face masks were mandatory, more than 95% of the older population had all their scheduled vaccines, and the entrance to restaurants or other indoor public spaces was limited to vaccinated people [35].

The intervention’s feasibility was based on the impact of the intervention on the participants’ well-being and the acceptability of the intervention by the participants in order to determine if the intervention was suitable for implementation on a larger scale or in different contexts. Therefore, to assess the impact of the BLOC project on the well-being of older individuals, changes in feelings of loneliness (including emotional and social loneliness), social support, anxiety and depressive symptoms, self-perceived health, and health-related quality of life were evaluated through telephone interviews before and after the intervention. The telephone interviews were conducted by two members of the research team, who had previously received training in administering item-based questionnaires. Intervention acceptability was assessed based on participants’ attendance and on a feedback survey.

### Ethics approval and consent to participate

This study was conducted in accordance with the ethical standards set forth in the Helsinki Declaration (1983). The protocol received Fundació Sant Joan de Déu (Barcelona, Spain) Research Ethics Committee approval (PIC-128-21). Individuals were included in the study only after giving their written informed consent.

### Participants

The research team contacted various primary care health centres and centres for the elderly in Barcelona to disseminate the study through informative posters, pamphlets, and calls. All the contacted centres were geographically close to encourage participants to stay in touch beyond the intervention. The dissemination material to attract participants called for people who wanted to connect more and better with others. Among the calls made from primary care centres and community centres, the attendees at senior centre presentations, and those who saw the advertisements, approximately 500 individuals with the appropriate profile were given the option to participate in the study. Out of these, 63 expressed interest and were screened starting in October 2021. The research team performed telephone screenings based on the inclusion/exclusion criteria for interested participants and those included in the study signed the informed consent form (n = 27) (see **S1 Fig**).

The inclusion criteria were as follows: (i) being ≥ 65 years, (ii) expressing the need to connect more and better with other people, (iii) wishing to participate, and (iv) having internet and computer/smartphone access. The exclusion criteria were: (i) being blind or deaf, and (ii) reporting cognitive impairment.

### Intervention

Participants were divided into groups of 6–8 participants and 2 facilitators. The facilitators of the intervention were all gerontologists with experience in psychosocial interventions with older adults. In each intervention, one facilitator was a psychologist, while the other was either a medical doctor or a sociologist. Groups met once a week for 8 weeks through the ‘Zoom’ videoconferencing online platform. Each session lasted between 90 and 120 minutes. The distribution between groups was based on participants’ schedule availability. Technological assistance by telephone and WhatsApp was provided to those participants who needed support to participate in the intervention.

**Fig 1:**
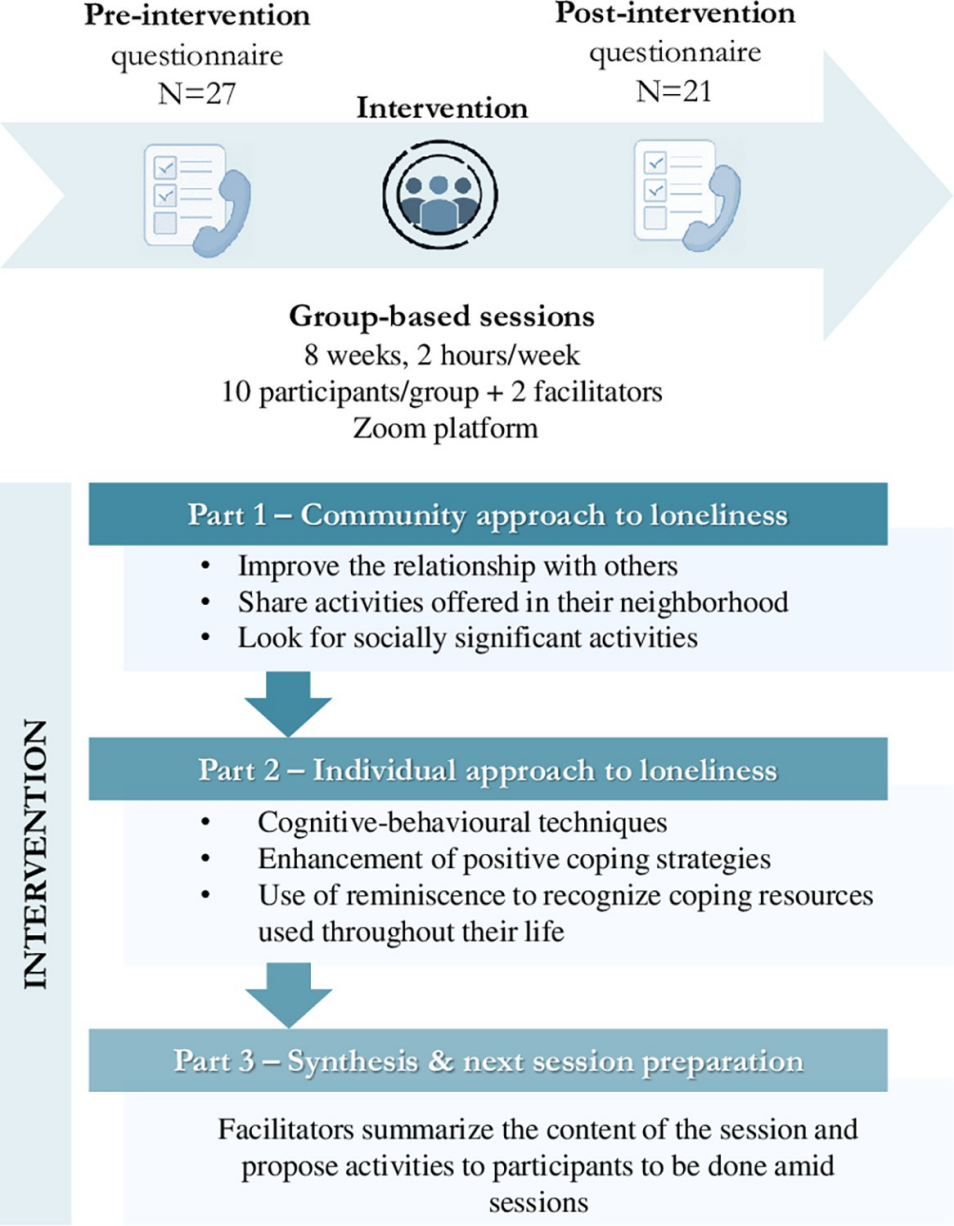
Scheme of the study design and sessions content of the group-based intervention.

Sessions were divided into two parts, each facilitated by one of the facilitators: (1) community approach to loneliness (i.e., activities to improve the relationship with others, learning about neighbourhood activities, and looking for socially significant activities); and (2) individual approach to loneliness through peer support (i.e., activities based on cognitive-behavioural techniques, enhancement of positive coping strategies, sharing experiences oriented towards the sense of purpose in life, and use of reminiscence for the recognition of coping resources used throughout the life cycle). At the end of all sessions, facilitators proposed activities to participants to be done amid sessions, which were linked to the next sessions’ topic. The last sessions dedicated some time to give continuity to the group once the intervention was over (**Fig 1** and **S1 Table**). The modular structure of the intervention allowed group facilitators to adapt the different sessions to the group needs and to the individuals who comprised it.

### Instruments

Outcome measures were assessed before and after the intervention through an interview, while sociodemographic data (i.e., age, gender, partner status, and educational attainment) were just asked before.

The primary outcome measure was loneliness, assessed through the 11-item De Jong Gierveld Loneliness Scale, obtaining a global score of 0–11, where higher scores indicate higher levels of loneliness [36]. This scale contains the social and emotional subscales. Responses in each subscale are summed to produce a score from 0 to 5 for social loneliness, and from 0 to 6 for emotional loneliness [37].

Secondary outcomes were social support, depressive and anxiety symptoms, quality of life, and health status. Social support was measured using the Oslo Social Support Scale (OSSS-3), which ranges from 3 to 14, with higher values representing stronger social support [38]. Depressive symptoms were measured on a scale of 3 to 24 using the 8-item Patient Health Questionnaire Depression Scale (PHQ-8) [39]. Anxiety symptoms were evaluated using the Generalized Anxiety Disorder Scale (GAD-7) [40], a 7-item measure with a total score ranging from 0 to 21. In both scales, higher values represent greater emotional disorder symptoms. We used the health-related quality of life questionnaire (EQ-5D-5L) [41, 42], which has two sections. First, the EQ-5D descriptive system was used to measure quality of life in terms of 5 dimensions: mobility, self-care, usual activities, pain/discomfort, and anxiety/depression. Second, the EQ visual analogue scale (EQ VAS) was used to evaluate one’s general health, ranging from 0 (i.e., the worst health you can imagine) to 100 (i.e., the best health you can imagine).

Finally, at the end of the study, during the post-evaluation quantitative interview, we included an additional open qualitative question. Participants were invited to provide any comments or feedback regarding the project with the prompt: "Please feel free to leave any comments or feedback about the project". This approach enabled participants to assess their satisfaction with the study and offer valuable insights.

### Data analysis

#### Sample size calculation

Based on a previous study [27] with a similar sociodemographic profile and outcome measurements, accepting an alpha risk of 0.05, a beta risk below 0.20 in a two-tailed test, assuming a standard deviation of 2.33, and considering a drop-out rate of 25%, using ANOVA, a minimum of 20 subjects were needed to detect a significant difference greater than or equal to 1.7 units in loneliness between pre- and post-measurements.

#### Statistical analyses

The characteristics of the study sample before (T1) and after (T2) the intervention were assessed using frequencies and proportions for categorical variables and medians and standard deviations for continuous variables. Differences between T1 and T2 were evaluated by applying the χ2-test for categorical data, and the Student’s t-test for continuous variables. Effect sizes (Cohen’s *d*) were calculated for the outcome variables based on the guidelines proposed by Cohen (1988) [43]: small effect size (d = 0.2), medium effect size (d = 0.5), and large effect size (d = 0.8). Cronbach’s alpha values were calculated to assesses the internal consistency of the main measurement tools (see **S2 Table**).

Mixed-effects linear regression models for repeated measures were constructed to study changes in the outcome measures (social and emotional loneliness, social support, depressive and anxiety symptoms, quality of life, and perceived health) between T1 and T2. Two-level random intercept models (“mixed” command in Stata) were fitted through maximum likelihood. The models used time point (T1 or T2) as a within-participant repeated factor and participant ID as a random factor. We assessed unconditional models to justify the use of hierarchical linear modelling (HLM) or mixed-effects regression. These models were constructed without any predictors to effectively partition the data into level 2 units. The results consistently showed significant random effects for the ID variable, which supports the adoption of mixed-effects models. By employing these approaches, we can properly accommodate the data’s multilevel structure and address temporal dependencies, thereby enhancing the robustness of our estimates regarding the effects of independent variables on the outcomes.

As statistically significant differences were found in the proportions of males and females between T1 and T2, the models were adjusted for sex. From these models, estimated means for the outcome variables were calculated through the margins command in Stata 13 [44]. All reported p-values were based on a two-sided test, where the level of statistical significance was set at p<0.05.

Stata version SE 13 [45] was used to analyse the data.

## Results

The study sample (N = 27) was mainly composed of females (74%) and the mean age was 74.26 years (66–88 years) (**Table 1**). Most participants had achieved a secondary education level (48%) and were married or in a relationship (33%). Significantly more women dropped out at post-intervention (*p*<0.05), while no significant differences were observed in the remaining sociodemographic variables. The means of the outcome variables improved from T1 to T2: quality of life, perceived health, and social support increased, while loneliness, and depressive and anxiety symptoms decreased. In the case of depressive symptoms and emotional loneliness, this improvement was statistically significant (*p*<0.05) with medium to large effect sizes (**Table 1**).

**Table 1:** Participants’ characteristics at baseline (T1, pre-intervention) and after the intervention (T2, post-intervention).

Variable	T1 (n = 27)	T2 (n = 21)	*p*-value^1^	*Effect size* ^*2*^
**Sex**, n(%)				
Male	7 (25.93)	6 (28.57)	0.0419*	
Female	20 (74.07)	15 (71.43)		
**Age**	74.26 (5.27)	73.90 (5.52)		
**Education**, n(%)				
Primary	4 (14.81)	3 (14.29)	0.4397	
Secondary	13 (48.15)	12 (57.14)		
Tertiary	10 (37.04)	6 (28.57)		
**Partner status**, n(%)				
Single	6 (22.22)	3 (14.29)	0.8217	
Married or in a relationship	9 (33.33)	8 (38.10)		
Widowed	7 (25.93)	7 (33.33)		
Divorced	5 (18.52)	3 (14.29)		
**Quality of life** (0–1), M(SD)	0.86 (0.16)	0.88 (0.13)	0.2511	-0.2580
**Perceived Health** (0–100), M(SD)	72.96 (16.48)	78.43 (12.98)	0.1343	-0.3406
**Social support** (3–14), M(SD)	10.48 (2.53)	11.05 (1.72)	0.6657	0.0957
**Loneliness** (0–11), M(SD)	4.33 (3.32)	3.19 (2.48)	0.3984	0.1883
• **Social** (0–5), M(SD)	1.70 (1.84)	1.76 (1.67)	0.1036	-0.3722
• **Emotional** (0–6), M(SD)	2.63 (1.96)	1.43 (1.33)	0.0180*	0.5622
**Depressive symptoms** (0–24), M(SD)	6.48 (4.95)	3.57 (4.02)	0.0087*	0.6350
**Anxiety symptoms** (0–21), M(SD)	4.37 (3.80)	3.29 (2.81)	0.1289	0.3457

NOTE: n = frequency; M = mean; SD = standard deviation.

^1^Differences were measured through chi-squared test for categorical variables and T-test for continuous variables.

^2^Effect sizes (Cohen’s *d*) were calculated based on the guidelines proposed by Cohen (1988) [43].

The estimated means presented in **Table 2** show statistically significant decreases in depressive symptoms and emotional loneliness (*p*<0.01). Emotional loneliness decreased by 0.84 points on a scale of 0 to 6, and depressive symptoms decreased by 2.30 points on a scale of 0 to 24.

**Table 2:** Estimated means of outcome variables in T1 (pre-intervention) and T2 (post-intervention).

Variable	T1 (n = 27)	T2: (n = 21)	*p*-value
**Quality of life** (0–1)	0.86 (0.80, 0.91)	0.88 (0.82, 0.94)	0.239
**Perceived Health** (0–100)	72.94 (67.25, 78.63)	76.82 (70.83, 82.81)	0.070
**Social support** (3–14)	10.49 (9.57, 11.41)	10.45 (9.51, 11.39)	0.840
**Loneliness** (0–11)	4.32 (3.19, 5.46)	3.86 (2.67, 5.04)	0.218
• **Social** (0–5)	1.70 (1.04, 2.35)	2.03 (1.34, 2.71)	0.128
• **Emotional** (0–6)	2.62 (1.98, 3.27)	1.78 (1.09, 2.47)	*p*<0.01
**Depressive symptoms** (0–24)	6.46 (4.79, 8.13)	4.16 (2.38, 5.94)	*p*<0.01
**Anxiety symptoms** (0–21)	4.36 (3.12, 5.60)	3.23 (1.87, 4.60)	0.104

NOTE: Estimated means calculated from mixed linear models including wave (T1 or T2) and sex as covariates. Means with 95% confidence interval are reported.

^1^*p-*values calculated with mixed models Wald tests.

Most of the participants used their own computer (59.3%) or their mobile phone (29.6%) to carry out the sessions. The link to the Zoom session was mainly sent by e-mail (66.7%) or WhatsApp (29.6%). The drop-out rate was 22%. The post-intervention interview was done to the 21 participants who had at least completed one session (81% assistance to ≥5 sessions).

Qualitatively, participants positively evaluated the intervention and found in the group a space for personal growth, where they could meet new people and express themselves with confidence and security. Most participants gave their group peers their phone number to keep in touch and some even met face-to-face to do social activities.

## Discussion

The ‘Breaking Loneliness, Opening Community’ (BLOC) pilot intervention aimed to promote the participants’ development of coping strategies to cope with feelings of loneliness while reflecting on the social meaning of loneliness in late life and, conversely, to increase social support by being a group-based intervention. Our study sample, on average, presented moderate levels of loneliness and mild depressive symptoms at baseline, which agrees with previous literature reporting their frequent co-occurrence [46–48]. Following the intervention, participants exhibited a decrease in loneliness, notably showing a substantial reduction in emotional loneliness (*p*<0.01). Furthermore, the statistically significant (*p*<0.01) decrease in participants’ depressive symptoms highlights the importance of an intervention that can reduce depressive symptomatology, especially given the increase in the prevalence of depression during the COVID-19 pandemic [49], which already placed a substantial burden prior to the pandemic.

The intervention had a high assistance rate for the majority of the sessions and a low drop-out rate, indicators that, together with the positive qualitative evaluation of the participants, reflect the acceptability and participants’ motivation to participate in the intervention. Females were more prone to participate in the study, which aligns with previous studies that have reported that among older adults, the digital gender gap has been compensated in recent years, and now females use more internet for social contact [50, 51].

However, contrary to what we expected, we did not obtain significant changes in the outcomes related to social relationships in an objective sense (i.e., increase in social support or a reduction in social loneliness). In the present study, to recruit participants, a call was made to those individuals who “wanted to connect more and better with others”, reflecting a desire to improve their social support at that time. Almost 60% of the sample reported loneliness, having a 44% of the sample moderate loneliness (de Jong score between 3–8) and 15% severe loneliness (de Jong: 9–11), reflecting a need to enhance their social relationships and highlighting a deficiency in either the individual or community dimensions of social interactions. Nevertheless, most participants (67%) had a moderate level of social support (OSSS-3: 9–11). This could suggest that participants likely have access to social support, and that difficulties in connection might more commonly stem from the qualitative or individual aspects of their social relationships. Our sample scored higher in the dimension of emotional loneliness compared to social loneliness at baseline, which aligns with previous studies showing a peak in emotional loneliness in older adulthood, while social loneliness is more stable across adulthood and drops at later stages of life [52, 53]. The significant decrease in emotional loneliness after the intervention could be due to the participants finding a confidence and intimate environment in the intervention group, where they gave each other support and understanding when sharing their thoughts and feelings. In this way, even though perhaps their social network was already satisfactory, they did not have the necessary closeness and emotional support that the group provided them.

The content of the intervention relied on the idea that we need to address the new needs derived from the pandemic and, at the same time, attend to pre-existing unmet mental health needs from before the pandemic. A significant reduction in depressive symptomatology was identified after the intervention, with the study sample going from having mild depressive symptoms before the intervention to minimal symptomatology afterward. Therefore, interventions providing peer support groups to combat loneliness and, at the same time, increasing the likelihood of establishing satisfying social relationships might help to reduce the burden of depression among older adults and reduce the significant economic costs associated with it [54].

### Strengths and limitations

The results of this study should be interpreted considering some limitations. First, the absence of a control group limits the possibility of attributing the results to the intervention. Second, the small sample size limits the statistical significance of our results. This was a pilot study with a pre-post design and with a short follow-up; therefore, the results should be treated as preliminary. A future study in a clinical trial format and a longer follow-up could allow its verification. Moreover, it is crucial to consider that the clinical or practical significance of these reductions may be limited by factors such as the specific characteristics of the study population. Future studies should explore interventions targeted at more specific inclusion criteria, which could offer insights into tailored approaches for achieving more substantial and meaningful outcomes in reducing loneliness and depression. Third, although technological assistance was available for those participants who needed it, this type of intervention may exclude individuals who do not feel confident in handling technologies and who do not have access to them. Finally, our data are based on self‐reported questionnaires, so reporting or recall bias could be present. Nevertheless, in our study, the recall periods were short and well-defined, which should minimize recall bias. In addition, acceptable internal consistency was found for the measures reported in our sample (see **S2 Table**), suggesting reliable measurements.

## Conclusions

Interventions overcoming social distancing restrictions through online tools and targeting vulnerable population sectors (e.g., older adults) can become essential to lessen the collateral consequences of the COVID-19 pandemic on social behaviour and mental health. The present pilot study tested a promising online psychology tool to reduce emotional loneliness and depressive symptoms, with a high rate of assistance to most of the sessions and a low drop-out rate. A future randomized controlled trial is needed to explore the impact of the present intervention on a larger sample of older adults.

## Supporting information

S1 Fig CONSORT diagram. (TIF)

S1 Table Contents of each session of the intervention. (DOCX)

S2 Table Cronbach’s alpha for the main measurements. Cronbach’s alpha (0–1) assesses the internal consistency of the measurement tools, with higher values indicating a higher agreement between items. A value of alpha between 0.70 and 0.95 is considered acceptable.
(DOCX)

## List of abbreviations

COVID-19: Coronavirus Disease 2019
MDD: major depressive disorder
BLOC: Breaking Loneliness, Opening Community
OSSS: Oslo Social Support Scale
PHQ: Patient Health Questionnaire Depression
GAD: Generalized Anxiety Disorder Scale
EQ-5D-5L: health-related quality of life questionnaire
T1: assessment before the intervention
T2: assessment after the intervention
