# Supplementary figures and images for: Reducing loneliness and depressive symptoms in older adults during the COVID-19 pandemic: A pre-post evaluation of a psychosocial online intervention
Source: PLoS One. 2024 Dec 13;19(12):e0311883. doi: 10.1371/journal.pone.0311883 (PMC11642987; doi:10.1371/journal.pone.0311883)

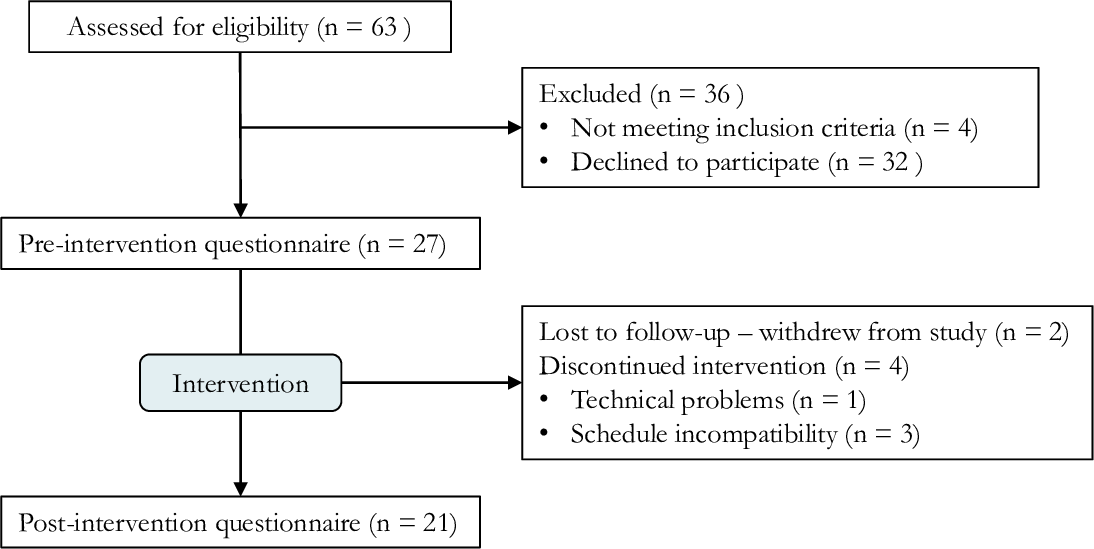

Supplement: S1 Fig — (TIF) [file pone.0311883.s001.tif]
