# Supplementary material for: Reducing loneliness and depressive symptoms in older adults during the COVID-19 pandemic: A pre-post evaluation of a psychosocial online intervention
Source: PLoS One. 2024 Dec 13;19(12):e0311883. doi: 10.1371/journal.pone.0311883 (PMC11642987; doi:10.1371/journal.pone.0311883)
Supplement: S1 Table — (DOCX) [file pone.0311883.s002.docx]

| **SESSION** | **PART 1: community approach to loneliness** | **PART 2: individual approach to loneliness** | |
| --- | --- | --- | --- |
|  |  | **During the session** | **Between sessions** |
| **1** | **Who are you?**  OBJECTIVE: each person in the group introduces themselves by showing and explaining a personal object. | **Unwanted loneliness. Approach to the issue.**  OBJECTIVE: Approach the recognition of one's own loneliness and the group as a space of equals. | Look for images or examples that we think represent loneliness as something positive or negative. |
| **2** | **Working with photographs.**  OBJECTIVE: share views and experiences about loneliness, social relationships, and participation in activities by commenting on photographs. | **Stigma and loneliness. Bringing it to light.**  OBJECTIVE: Understand how loneliness has social roots and review causal attributions (from exclusively internal to external; from blame to social responsibility). | Recognizing the moments when we feel lonely in our daily life and when we feel better. |
| **3** | **What would you like to do?**  OBJECTIVE: share topics and hobbies of interest among the participants. | **The impact of loneliness. The consequences of loneliness.**  OBJECTIVE: Become aware of the impact of loneliness and identify the most affected areas to set goals. | Think about what we have learned throughout life that can help us cope with loneliness. We think of phrases or ideas that help us in the toughest moments. |
| **4** | **Our neighbourhood**  OBJECTIVE: share knowledge and experiences about the opportunities and barriers in the neighbourhood for participating in activities. | **Loneliness throughout life. Tools in the backpack.**  OBJECTIVE: Review one's own life cycle to place loneliness in the personal narrative as something that happens throughout life and value the accumulated experience. | Think about what we do when we feel lonely and how our life should be so that we do not feel lonely (we seek realistic and practicable goals). |
| **5** | **What do you want to change?**  OBJECTIVE: Set short-term goals to make behavioural changes according to the preferences and priorities of the participants and the available resources. | **Everyday resources. How we can feel better in daily life.**  OBJECTIVE: Identify and work on the resources generated by each participant, assess their potential impact, and foster group commitment. | Put these resources into practice. |
| **6** | **Short-term goals**  OBJECTIVE: Thoroughly review the achievement of the proposed goals. | **Proximity resources. Doing and knowing how to do it (or not).**  OBJECTIVE: Share social activities of the past weeks and how difficulties have been or should have been faced. | Put these resources into practice (and/or the previous ones, session 5). |
| **7** | **How can we continue?**  OBJECTIVE: Give continuity to the group once the intervention is over. | **Looking to the future. Aging in loneliness.**  OBJECTIVE: Promote positive coping with possible changes. | Think about the most significant learnings from the sessions we have conducted and the personal challenges that lie ahead. |
| **8** | **We finish and continue**  OBJECTIVE: Briefly review the achievement of the proposed goals. Closing the space. | **Group learnings and commitments.**  OBJECTIVE: Become aware of the group's learnings and the contributions each person has made to the others, recognizing the participants and enhancing their self-esteem. |  |

*.*
