# Supplementary material for: Reducing loneliness and depressive symptoms in older adults during the COVID-19 pandemic: A pre-post evaluation of a psychosocial online intervention
Source: PLoS One. 2024 Dec 13;19(12):e0311883. doi: 10.1371/journal.pone.0311883 (PMC11642987; doi:10.1371/journal.pone.0311883)
Supplement: S2 Table — Cronbach’s alpha (0–1) assesses the internal consistency of the measurement tools, with higher values indicating a higher agreement between items. A value of alpha between 0.70 and 0.95 is considered acceptable. (DOCX) [file pone.0311883.s003.docx]

| **Variable**, measurement scale | *Cronbach’s alpha* |
| --- | --- |
| **Quality of life**, EQ-5D | 0.6647 |
| **Social support**, OSSS-3 | 0.8036 |
| **Loneliness**, de Jong | 0.8427 |
| - **Social** | 0.8432 |
| - **Emotional** | 0.7409 |
| **Depressive symptoms**, PHQ-8 | 0.7378 |
| **Anxiety symptoms**, GAD-7 | 0.7452 |
